# Business models for the Anthropocene: accelerating sustainability transformations in the private sector

**DOI:** 10.1007/s11625-021-01037-3

**Published:** 2021-10-05

**Authors:** Sarah Burch, Jose Di Bella

**Affiliations:** University of Waterloo, Waterloo, Canada

**Keywords:** Anthropocene, Sustainability, Business models, Experimentation, Development paths, Transformation

## Abstract

The rapid pace and escalating severity of climate change impacts have made clear that current incremental approaches to pressing global socio-ecological challenges are insufficient to address the root causes of unsustainable development. This has spurred increasing interest in the dynamics of transformation: the actors, capacities and resources needed to fundamentally shift development paths. The private sector is at the core of essential transformative processes necessary to build a future premised on environmental integrity, social inclusivity, and resilience. The activities of the private sector are structured and driven by their underlying business model, which is at its core a set of assumptions about how a business creates, extracts and delivers value. Dominant conceptualizations of the business model remain a narrow imagining of how business interacts with societal processes and shape development patterns. In this article we call for the conceptualization and design of business models anchored in societal purpose and operating within planetary boundaries, apt for the Anthropocene. We identify five building blocks for business models where transdisciplinary sustainability research can accelerate entrepreneurial activity that fosters desirable sustainable pathways by enabling the creation of new capabilities in support of broader transformational processes. This article seeks to inform (and potentially re-orient the efforts of) transdisciplinary scholars engaging the private sector in the co-production of community-based sustainability and resilience-building initiatives. Likewise, the building blocks provide a guide for businesses who aim to deepen their capacity to build new partnerships, identify, and incorporate new information on climate risk into their operations and develop practices, sequences and procedures oriented toward the sustainable development goals and disaster resilience.

## Introduction

The anthropogenic cause and dramatic but unequally distributed impacts of global climate change have been both observable and well-studied over the last two decades (IPCC 2007; Stocker et al. 2013; King et al. 2015). These global dynamics, however, create deeply local challenges: place-based socio-ecological dynamics fraught with unparalleled challenges for governments, organizations and individuals that will require a rapid transformation toward more sustainable development pathways (Folke et al. 2020). Society, however, is still coming to terms with the limits placed on the economy by the biosphere, a premise largely ignored by foundational principles of contemporary economic models, constructed on deeply held assumptions about technological progress, consumption and endless GDP growth. The dominant modern form of capitalist system, furthermore, which continues to result in higher levels of inequality, deepening the climate crisis and rapidly eroding ecosystems (Stiglitz 2021; Beer 2020; Wolfgang et al. 2016; Beer 2020; Pelling and Navarrete 2012) is generating social unrest and political tensions in countries around the globe.

Multiple, intersecting climatic risks present significant challenges for protecting hard-earned developmental gains, making ever more pressing the need to deepen societal resilience and address developmental gaps. Widespread climate change impacts (Schleussener et al. 2016) and the halting, scattered progress toward deep decarbonization suggest that incremental actions are insufficient to respond to the growing societal demands to build sustainable and resilient communities. Solutions that are focused exclusively on technology are likewise insufficient (Burch 2010; Burch et al. 2014), neglecting the underlying worldviews (Hulme 2009), complex economic architecture, and fractured politics that characterize what is now widely called the Anthropocene (Steffen et al. 2011). Only a profound shift in the configuration of growth-oriented models (Nightingale et al. 2020) can begin to address the root cause of risk: the unsustainability of current economic patterns, and an extractive relationship to nature. Indeed, new processes and capacities must emerge that feed a fundamentally altered relationship between humans and the environment, driven by visions and stories of what a desirable future might look like (Bennett et al. 2016; Pereira et al. 2018a, b).

The idea of transformation, which refers to profound changes in societal values, beliefs, and practices at the individual, organizational, and broader social scales (O’Brien 2018), has been at the forefront of the most recent policy and academic debates around sustainability (Burch et al. 2014; Feola 2015; Abson et al. 2017; Elmqvist et al. 2019). The depth and breadth of change necessary to build a society that provides long-term well-being, prosperity, and environmental integrity, will require the social reconstruction and transformation of the most widespread type of organizations: privately held, for-profit firms.

The activities of the organizations that comprise the private sector are structured and influenced by their underlying business model, which is at its core a set of assumptions about what a business will and won’t do to create value. The vast majority of these private actors operate under business models that focus almost exclusively on bottom-line value-generating mechanisms (Osterwalder et al. 2005). There have been efforts to promote changes to private sector functions to widen their contribution to social and environmental integrity through sustainable business models (Wagner 2012; Schaltegger et al. 2016; Evans et al. 2017). Still, a gap remains in examining not just the function but the underlying purpose of these organizations, which is critical to understanding how we might deliver desirable and necessary changes both to the nature of these organizations, and their capacity to offer sustainable solutions. Cultivating climate-resilient and sustainable communities requires a radical reconfiguration of private sector organizations’ architecture, mechanisms and objectives (Few et al. 2017) to accelerate the adoption of sustainability-oriented thinking and actions, but also to interrogate their “*raison d'être*” in social life.

The business model is the configuration that dictates the use of resources, aims of partnerships, degree of disclosure of information, the considerations in the selection of raw materials and inputs, and value-generating tools to achieve the designed goals—but most importantly, their behavior in the pursuit of profits (Baden-Fuller and Morgan 2010; Teece 2010). A widely held paradigm in the private sector, that the “only social function of business is to create value to its shareholders” (Friedman 1970), has been the foundation of business models over the past five decades with many operational and strategic implications, but also with inherently social, environmental and political consequences.

While these forms of profit-seeking configurations are ubiquitous, emerging research shows that small- and medium-sized enterprises (for instance) are social and political actors (Westman et al. 2020), not purely rational economic ones. Furthermore, these important actors, which might employ up to 500 people, generate millions in revenue and have hundreds of supply relationships can have significant influence in shaping local policy and make strategic contributions to trigger systemic changes necessary to steer communities towards the type of sustainable development pathways (Rosenzweig and Solecki 2018) needed to find a balanced relationship between society, economy and the planet.

While socially and environmentally oriented business models have begun to emerge, bringing new types of enterprises into the organizational landscape, and offering novel ways to attain and deliver value compatible with sustainability targets, they remain relatively rare. These types of initiatives have led to a renewed interest in the concept of the business model beyond a purely managerial term, but also as the entry point for research and global policy circles to leverage the resources of the private sector in support of progress toward, for instance, the Sustainable Development Goals, the Sendai Framework for Disaster Risk Reduction and the Paris Agreement.

While arguments have arisen in favour of making unique and more deeply sustainable business models the norm in the future (Business and Sustainable Development Commission 2017), the efforts to replicate sustainable businesses models places the bulk of the effort on promoting ‘shared value’ practices to enhance the private sector contributions to sustainability—arguing for a win–win scenario in which profit is delivered alongside environmental or social value.

This transactional approach to leveraging private sector contributions to sustainable development, however, neglects the deeper transformation in a firm’s purpose, and its position within an evolving social and environmental landscape. In contrast to an approach that simply achieves greater resource use efficiency, for instance, this deeper focus on purpose requires a relational, systems-oriented view of the firm (DiBella 2018).

## Looking outward from the firm to its local context: a relational view

In practice, departing from a firm-centric approach (Freeman and McVea 2005) towards a relational perspective requires establishing clear linkages between the socio-ecological context of enterprises and the organizational architectures, rationales and values that underpin them. Businesses can incorporate and share local knowledge for adaptation (Biagini et al. 2014), plan long-term carbon reduction investments with partners (Britton and Woodman 2014), participate in sustainability and resilience-oriented efforts of local municipalities (Burch et al. 2013), and contribute to the creation of circular economy practices (Lüdeke-Freund et al. 2019). These examples begin to sketch a roadmap for the future of sustainability efforts in the private sector with a new purpose that individual enterprises, owners, managers, and employees can craft in the era of the Anthropocene.

The researchers and practitioners working with businesses and seeking to advance sustainability can support the construction of a distinct organizational landscape with the potential to deliver a desirable future in a ‘good’ Anthropocene (Pereira et al. 2018a, b; Bennett et al. 2016) by broadening the understanding of the business model as a unit of analysis. Framing the business model as an activity system opens the opportunity for businesses to strategically engage at distinct leverage points (Meadows 2008), such as how power is distributed by changing hierarchies, limiting or encouraging different feedback loops, and promoting new and usable forms of information flows to influence and accelerate desirable local changes. A novel set of building blocks for private sector organizational structures can unlock the potential siloed within larger organizations (Hall and Wagner 2011), which will require new approaches and methodologies to begin recognizing the many possible leverage points for making strategic contributions to sustainability transformations.

## Business models as activity systems

The business model is a system that structures the relationships, processes, assets, and physical objects as well as the value-generating functions of enterprises (Chesbrough 2010). Over the last decade, sustainability scholars have presented evidence of bottom-line models' evolution to more sustainable business models. These describe activity systems (Zott and Amit 2010): archetypes for private sector activities determined by their value propositions for sustainability (Böcken et al. 2014) or problem–solution patterns (Lüdeke-Freund et al. 2018).

**Fig. 1:**
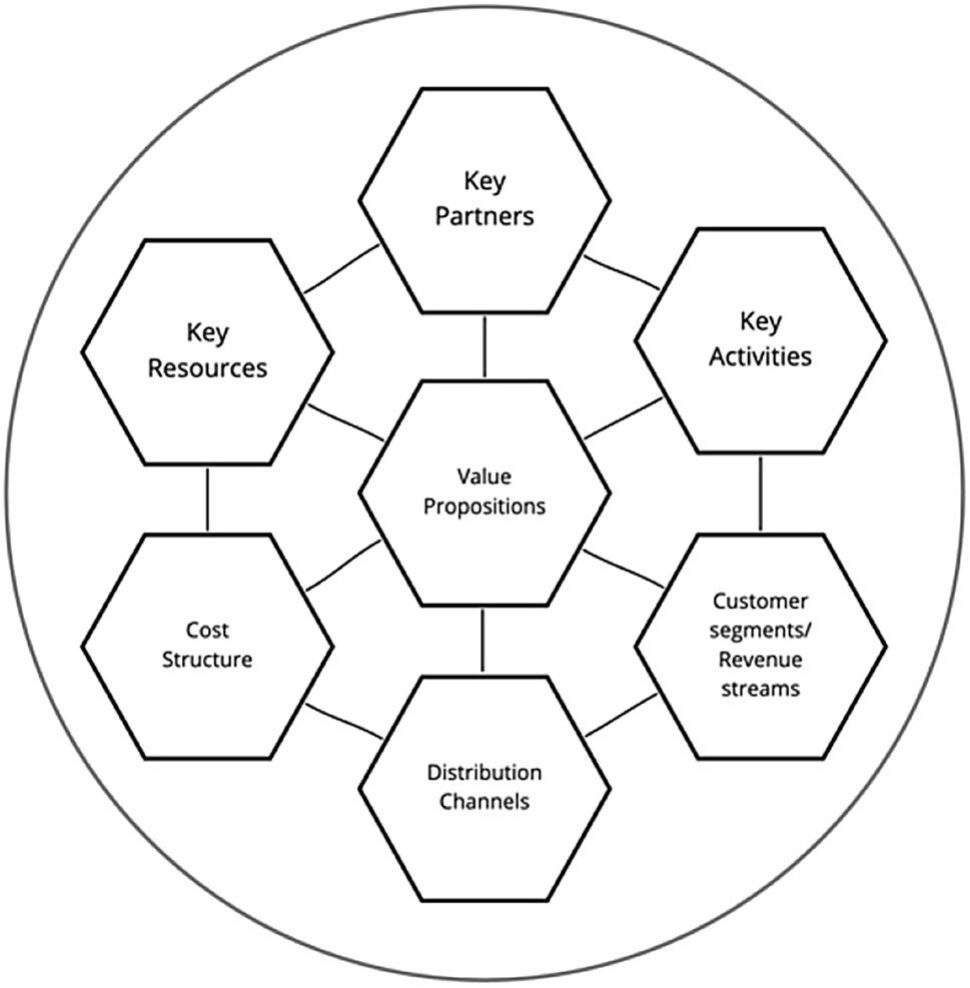
Adapted from the business model canvas (Osterwalder and Pigneur 2012)

Emerging private sector organizations with unique social and environmental value-generating configurations include cooperatives, b-corporations, and for-benefit corporations (Geissdoerfer et al. 2018), which have been part of recent sustainable business model studies. These organizations clearly articulate individual and specific contributions to sustainability (Stubbs and Cocklin 2008). However, most individual businesses’ fundamental structure has been described through its basic building blocks of activities, costs, customers, and logistics (Osterwalder and Pigneur 2012), which provide a static view of enterprises' value configuration (see Fig. 1) for a schematic of this view, and Table 1 for a range of business model framings in the literature).

**Table 1:** Summary of the most common ways to conceptualize business models, including as templates, as patterns, and as systems

Business model (BM) characterization	Examples of authors/contributions to conceptualizing BM
As templates	A matrix with the main components of an organization is used to describe the BM. This provides a template for organizations to identify and codify their value propositions, supply channels, and processes of profit-making mechanisms in the firm (Osterwalder et al. 2005)
	A template for conceptualizing sustainability to inform the firms decision-making processes and a tool to pinpoint where organizations must develop new organizational and culture capabilities oriented towards sustainable outcomes (Stubbs and Cocklin 2008)
	For developing strongly sustainable business model propositions driven by a framework of strongly sustainable business model propositions and principles to enable a description of the BM in terms of sustainability (Upward and Jones 2016)
	That provides a tool to entrepreneurs to explore the market and contribute to the construction of the techno-economic network of an innovation (Doganova and Eyquem-Renault 2009)
As patterns	Organized in a taxonomy of 45 patterns related to ecological, social, and economic value creation that support sustainability-oriented business model innovation (Lüdeke-Freund et al. 2018)
	To design and identify strategy options to support the closing of resource flows in businesses leading to circular economy patterns (Lüdeke-Freund et al. 2019)
	Or archetypes of mechanisms that inform innovation process for embedding sustainability practice in businesses and to help deliver sustainable outcomes (Bocken et al. 2014)
As systems	Represented in models much like those used in biology and economics, which can become instruments and sites for scientific inquiry (Baden Fuller and Morgan 2010)
	Activity systems of interdependent activities within and beyond the focal firm boundaries outlining a conceptual approach to finding a perimeter of organizational activities that have different design element and themes (Zott and Amit 2010)
	That can be understood through a spatial representation to pinpoint the processes and feedbacks of value generation and distribution, knowledge and flows of resources using an example of BM potential to establish a shared language among research and businesses, and to map climate-related risks in different geographies (DiBella 2019)

The figure provides a view of the main components of a business model. The approach to understand value creation and delivery in the firm-centric approach is anchored around how to manage those elements and relationships to support business activities and financial value creation.

A relational activity system begins to emerge through precise and scaled characterization of business operations’ practices and outcomes. A transformative business model conceptualizes value generation within and without the organization itself, recognizing the firm as an economic agent inextricably linked with broader capital development and investment processes (Schoenberger 2002) and as a social and political actor shaping and being shaped by local agendas, practices, and cultures (Westman et al. 2020). This type of model will account for the constraints of the social-ecological system and align its operations or resources to accelerate sustainability and resilience-building.

The possible leverage points (Meadows 2008) to support shifts in this activity system might be geographically dispersed in a multi-scalar system. For example, value-generating business components such as sourcing and logistics might have to account for climate risks in remote areas. Still, sustainability-oriented solutions can create social value, for example by supporting community partners to climate-proof operations, or by building partnerships to pursue or transfer sustainable practices.

Developing a better understanding of a business’s perimeter (and the porosity of that perimeter) is a fundamental step towards leveraging their resources and steering business models towards more strategic contributions to sustainability. Some efforts to map business models’ boundaries include spatial conceptualizations (DiBella 2019), which highlight the geographic distribution of business configurations where an array of relationships, knowledge, and resources interact to create value and represent possible interventions points to enhance sustainable outcomes.

In this paper we advance this emerging discourse by developing a conceptual framework that expands the notion of a business model to reflect the inter-relationships between firms and their broader socio-ecological landscape, the importance of more inclusive and creative organizational structures, and the challenges presented by pervasive, deeply path dependent (Westley et al. 2011) economic structures rooted in the primacy of profit.

## Methods

Our objective is to develop a conceptual framework that reveals the ways a business model might expand to build the new capabilities required to respond to the challenges presented by the Anthropocene. The emerging lessons from sustainability and climate research, and the underpinning societal challenges expressed in the existing climate, sustainability, and disaster risk policy frameworks suggest where the private sector should make robust contributions. The document analysis of peer-reviewed publications, recent policy (See Annex 1) and research agendas (Bradbury et al. 2019; Fazey et al. 2018) generated data that led to interpretation and understanding (Corbin and Strauss 2012; Bowen 2009) of some of the gaps in existing business model capabilities that might explain limited uptake of sustainable models.

Furthermore, we collected data from 65 small- and medium-sized enterprises to illustrate unique approaches to delivering social, economic or environmental value. We explored case studies of sustainable enterprises and their different sustainability-oriented practices and business models through a targeted search of peer-reviewed articles, grey literature (such as policy reports and monitoring/evaluation findings), and online cases nested in different studies (Flyvbjerg 2006) or organizational websites.

Finally, a preliminary database matrix containing information on sustainable enterprises and an array of practices was prepared, which helped to validate the set of building blocks that respond to the gaps identified in business model conceptualizations, but which are critical to address the key sustainability and resilience challenges. This analysis illuminated a trajectory from their description of simple financial value structure to the recent sustainability business model categories (Morris et al. 2005), patterns (Lüdeke-Freund et al. 2018) and ontological categories (Upward and Jones 2016).

## Foundational building blocks of business models for a good Anthropocene

The formation of new capabilities for coping with climate change impacts and delivering sustainability solutions at a pace and scale necessary to solve the Anthropocene’s local challenges requires a building a new organizational landscape. This requires means enabling new forms of businesses to emerge and flourish, which are capable of weaving complex social and environmental dimensions into the organization’s everyday interactions, shaping the organization’s purpose in communities and accelerating local change without losing their ability to be profitable enterprises.

The following building blocks focus on the mechanism needed for business firms to develop capabilities to address some of the priorities outlined in recent science and policy agendas, aimed at building sustainability and resilience into urban systems. These building blocks address foundational gaps in existing business models to steer businesses into making strategic and informed interventions to support the new pathways.

**Fig. 2:**
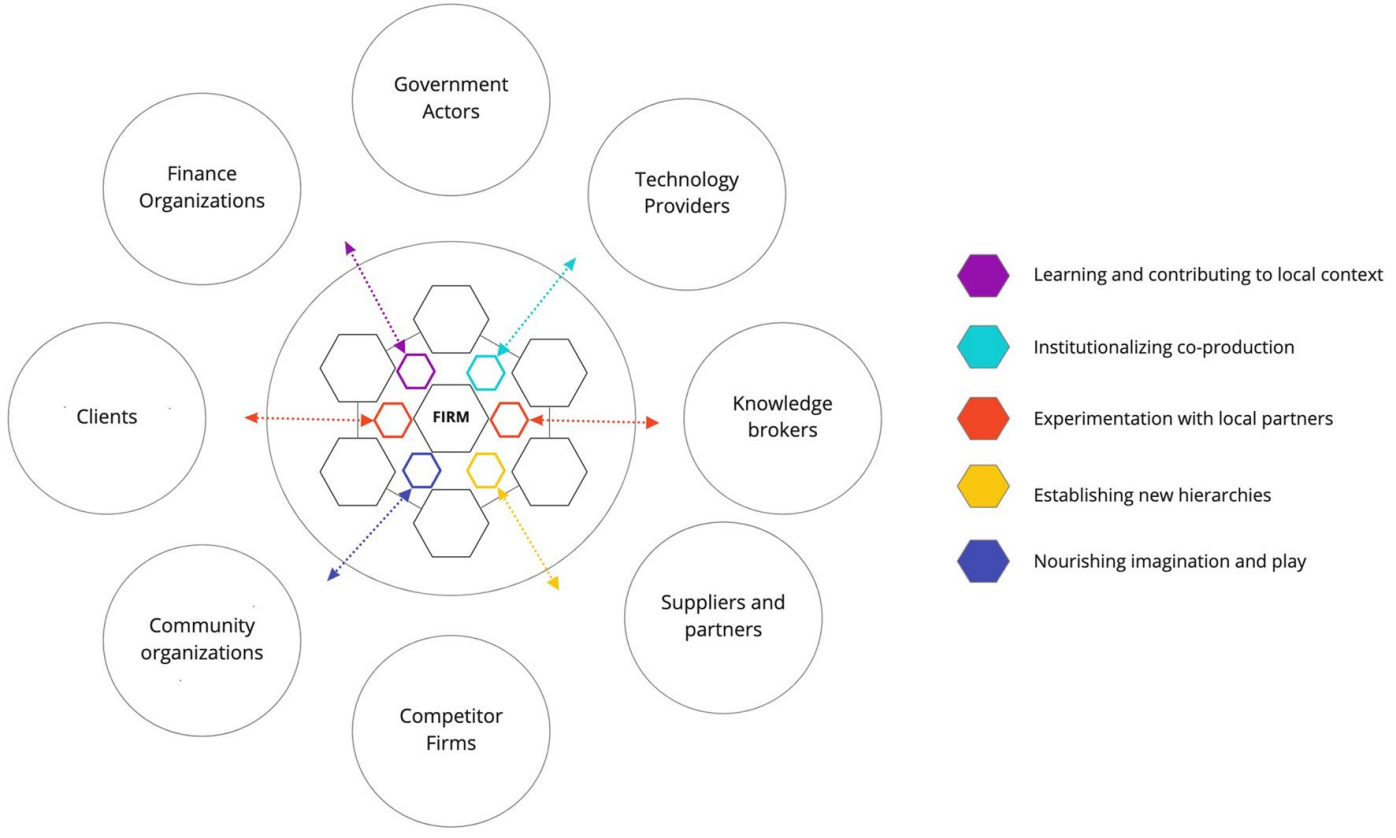
A transformative model based on building blocks which enhance the relational capabilities of an SME to influence system leverage points. Building blocks mediate the relationships between the firms’ elements and their external partners, community, and environment

We propose that these blocks (See Fig. 2) open avenues for businesses to shift into transformative business models. These would begin reconfiguring operations to accelerate sustainability transitions and build placed-based resilience (Grenni et al. 2020). Their integration into business structures requires institutionalizing these elements by establishing new organizational processes, assigning human resources, creating incentives, and making fundamental changes to the organizational architecture to enable their practice.

Sustainability science can contribute insights that help to accelerate bottom-up, fundamental, and non-linear shifts in the private sector, enabling system changes within and without the organization across crucial points in the activity system. Transformative models based on these building blocks enhance the relational capabilities of a business to influence system leverage points. Figure 2 builds on the static view of the firm and illustrates the ways that organizations are part of an activity system, where the building blocks mediate the relationships between the firms’ core elements and their external partners, community, and environment. A transformative business model is an activity system with the enterprise as the starting point, but with a complex array of relationships and capacities that decision makers and planners can begin to recognize or target with policy, and which have previously not been considered–or solely accounted for as externalities.

In the sections that follow, we outline each of these building blocks with a particular focus on the capabilities required to build business models that are responsive to the place-based demands the Anthropocene.

### Contributing to, and learning from the local context

To identify and pursue context-specific priorities, firms must establish mechanisms that recognize and build upon the deep and complex connections between the firm and their local environments. Implementation of strategic forms of collaboration and closer engagement with community actors is central to building transformative capacities within these organizations. Cultivating a stronger sense of place and context-specific values can drive local action and place-based sustainability transformations (Grenni et al. 2020), while also offering opportunities to reflect on (and even challenge) constraints on change at the finer scales that are presented by the inertia, lock-in, and complexity at other geographic and institutional scales. This building block calls for developing robust decision-making tools and processes within businesses to assess and incorporate climate risk information, and select suppliers and partners based on the shared pursuit of sustainable practices (Burch et al. 2019).

This contextualization, rooted in a deeper understanding of local needs, may become manifest in actions such as enhancing inclusive hiring practices or better equipping the organization to participate in the co-creation of place-based sustainability activities with civil society, local governments, or community partners. For example, a sustainable tourism company in Mexico has built an organic crop garden and edible forest for the local community and developed a sustainable tourism training program for residents to be part of a local conservation effort. These efforts are driven by an understanding of the local context and a deliberate effort to contribute beyond ecotourism, as well as a concerted conservation effort by a business.

This may also involve the allocation of time and effort to incorporate new knowledge that is seemingly unrelated to the firm’s core business model. For example, firms may expand their ability to share information relevant for adaptation planning with local business associations or provide their expertise for climate planning with local initiatives. For example, in Nicaragua, a chili agro-exporter company with a local supply chain participated in the Regional Project for Adaptation to Climatic Change for the Corporate Sector and sought to actively engage in improving the regional enterprise strategies for climate change in Central America (UNFCC Private Sector Adaptation Database). This is a fundamental step towards businesses better understanding the multiple dimensions of socio-ecological sustainability and magnitude of risk at the local level.

The COVID-19 pandemic and the increasing magnitude of multiple and intersecting disaster impacts on communities have highlighted that SMEs can be disproportionally affected by human-made or natural hazards. These organizations experience direct losses and increasing their challenges for meeting minimum operational requirements. A key goal for disaster risk reduction is understanding risk, which requires new partnerships, such as working with local weather agencies to provide usable data for decision making (Partey et al. 2019) or research partnerships with local institutions to quantify and map sources of disaster risk in business operations (Sarmiento et al. 2015) and across supply chains (Canevari-Luzardo et al. 2020) and seeking complementary capabilities, such as analysis of biophysical risk in specific locations, scenario building and engagement in community resilience building initiatives by working with community organizations engaged in disaster risk reduction. Business firms will need to begin undertaking substantial business model innovations to significantly shift organizational processes and change decision-making tools that can support community engagements for sustainable and resilient building processes.

Businesses require a more nuanced understanding of the local context to be able to adequately process information on the many social, economic, and environmental variables shaping a community’s degree of sensitivity to climate and disaster risk and using the information to develop apt disaster risk reduction and climate adaptation practices.

In a practice, these operational shifts require understanding employees’, suppliers’, and partners’ local conditions of risk, mobility challenges, disruptions, and challenges to livelihoods presented by climate stressors. These dimensions are not routinely considered but would enhance the potential for resilience-building activities driven by businesses. Their engagement in the local context would focus on strategic contributions such as place-making activities to promote coordination and interventions to support the city’s broader sustainability efforts (Burch et al. 2020). Furthermore, learning from the local context would facilitate partnerships to work with other local businesses to undertake risk reduction activities and work towards small but significant ecosystem-based contributions for local adaptation efforts.

In areas of climate and disaster risk, transdisciplinary approaches can help businesses find ways to more effectively synthesize and deploy climate risk information (Daniels et al. 2020). Identifying local practices to manage climate risks and working actively to help reduce the vulnerability of individuals, households, other organisations, or the place where their business is located, requires a broader understanding of the sources of risk, and how a systemic approach to building societal resilience includes the enterprise (Williams et al. 2019). A firm’s capability to learn from the local context, and in turn make contributions to that context, can be enhanced by researchers in developing “useful and usable information” (Lemos et al. 2012), drawing on multiple sources of knowledge and translating data into business language that can be used by firms to make more informed decisions.

These efforts are not solely limited to resilience-building but to more comprehensive approaches to tackling sustainability challenges that might support long-term viability of the organizations as a business entity and the capacity of their partners, employees, and communities to make real progress on developmental goals.

### Institutionalizing co-production

While participatory methodologies and action research facilitate change in communities of practice (Schneider et al. 2019b), the deployment of co-production mechanisms to advance inclusive governance processes (Turnhout et al. 2020) has yet to meaningfully engage with small businesses (Westman et al. 2020). The private sector’s role remains obscure in these activities, often plagued by limited transparency or barriers presented by the lack of apparent value for the enterprise, the preservation of proprietary information, and the drive to maintain competitive advantage. Furthermore, the pursuit of sustainability is a contested, multi-level governance challenge (Ehnert et al. 2018; Newig and Fritsch. 2009), in which shifting constellations of actors, with unevenly distributed power and capacity, navigate multiple, intersecting issue areas. Small firms are rarely able to participate directly in the coalition-building or lobbying efforts that might lead to more supportive national or regional (state/provincial) policy, due to the high transaction costs associated with these activities and immense coordination challenges (Schroeder et al. 2013). Reframing these barriers in light of cooperation to shared problem solving practices in an era of climate impacts and increasing societal pressure to address sustainability challenges requires the establishment of inclusive and iterative processes for business firms to collaborate openly in pursuit of public goods with partners, for example, in the formulation of regulations around energy or water efficiency, new technologies or targeted investments into productive assets or technology services.

The “occasional engagement” approach to collaboration remains squarely in the domain of firm-centric business models. A relational perspective calls for sustained participation, integration of processes, metrics (Durose et al. 2018) and assignment of internal champions in the organization to lead on the coproduction process with external partners. This more transformative approach to collaboration requires developing the capabilities and freedom for individuals to identify solutions–which often is a result of their business routines–but also to have space and resources to engage in a genuinely collaborative endeavor to solve specific problems associated with routine activities.

SMEs will need to build knowledge of how their operations link to broader urban systems to transition into sustainable models. These knowledge forms include technical, scientific, and local expertise to make community-based or local sustainability action part of their operations’ everyday routines. Institutionalizing co-production represents a shift from the SMEs’ efforts to develop well-established and linear business plans and create a mechanism within the organization that operates and links to its various parts. An organic agriculture company in the US, for example, has established a climate business unit that works directly with individual farmers along their supply chain in Mexico to co-produce climate and sustainability-oriented local solutions with individual partners, such as managing food waste, reduce disaster risk in flood and hazard zones. The initiative is actively co-producing and localizing sustainability-oriented solutions, replicating good practices, and collaborating to build local resilience. This business-driven mechanism has created a new unit that works with farmers and community members to document, test and replicate these practices based on a collaborative model aimed at replacing international certifications and creating an institutionalized avenue for information flows from the local partners to the business decision making processes.

The role of local firms in sharing costs and investing in this scale of local infrastructure, or supporting restoration projects, will require collaboration to develop shared solutions with government, community members and the other businesses. It is important to recognize, however, that without more effective coordination mechanisms, few firms will be capable of engaging in this process. Likewise, without specific attention being paid to systemic marginalization and unequal distribution of power among the “co-producers,” the benefits of sustainability transitions may also be unequally, and unfairly, distributed. Co-production does not erase these imbalances, but does provide an opportunity to reveal and potentially address them (Turnhout et al. 2020). Institutionalizing co-production practices within the organization would facilitate engaging in these decision-making processes by developing adequate organizational processes, tools, and guidelines, and establishing incentives for employees to engage in activities with external partners.

Institutionalizing co-production in meaningful ways can give voice to traditionally marginalized knowledge systems and actors (Burch et al. 2019; Latulippe and Klenk 2021). Transdisciplinary scholars working with SMEs can develop strategic approaches (Schneider et al. 2019a; b) to enable a culture of co-production in businesses by including different levels and types of employees, inviting external participants, and navigating the complex process of incorporating many ideas and experiences into the design of sustainability initiatives and projects (Reed and Abernethy 2017) with the aim of helping these efforts become central ways in which businesses approach sustainability efforts.

Creating new models for governance that support sustainability and resilience objectives is crucial for accelerating progress toward more resilient future pathways. We continue to observe in cities how the private sector might negatively influence decisions that impact the built environment and public space, for example leading to gentrification or controlling zoning regulations that affect biodiversity, green space and water use (Romero-Lankao et al. 2018; World Climate Research Program 2019). These examples illustrate the importance of establishing genuine coproduction mechanisms aligned to business model drivers to inform business strategy and practice to a shared sustainability vision.

### Experimentation with community partners, and an openness to failure

The capabilities emerging from experimentation result in building unique skillsets, testing new ideas, expanding the identity of the organization, and accepting the inevitability (and value) of failure. New ideas can emerge from interactions with community actors, through direct engagement but also through the design of activities aimed at inviting specific individuals or organizations to collaborate with the firm. This type of practice can be narrowly limited to research and development for new products or services, but can also include fostering new knowledge, collaborations, relationships and networks (Fuenfschilling et al. 2019). But strategic engagements with more diverse methods of participation in sustainability initiatives (França and Trygg 2017) or adoption of new technologies that enhance employees’ ability to better understand the impacts of the business can improve tactical decision-making that contributes to sustainability.

Significant potential exists to leverage the capabilities that are built through the iterative process of success and failure that is routinely associated with experimentation (Sengers et al. 2019). Existing business model of Fab Labs or maker spaces (for instance) engage citizens in a way that provides access to tools, resources, and ideas in a shared collaborative physical space (Edwards and Bulkeley 2018). Ultimately, these cases offer essential lessons for organizations on how to spur innovation through local cooperation and experimentation, engaging diverse communities of practice. For example, a local private primary school in Mexico City built an urban garden with solar power, developed in collaboration with a local community association. While the scale remains small, the mechanism of co-design and collaboration with local organizations pursuing sustainability goals led to local partnerships. These sustainability partnerships are not new for the private sector, but still are largely in the sphere of large corporations and international development organizations or initiatives. Transdisciplinary scholars can test new methodologies and help private sector organizations map, identify, and work with local partners to advance unique sustainability objectives in communities.

The building blocks of experimentation that commonly exist within SMEs include routine innovation, research, and development processes. However, experimentation beyond product or service development has not been widely explored. While the capabilities of testing and adapting new tools for improving efficiency reside in most organizations’ business models, fostering experimentation as a form of engagement with sustainability objectives and the climate change imperative remains a challenging activity for SMEs. This specific type of experimentation, however, will be a foundational activity for SMEs if they are to find ways to adopt deeply sustainable practices and understand social and environmental value from a more expansive system perspective, beyond the confines of their organizational thinking.

The examples of creative and transformative urban labs (cf. Voytenko et al. 2016) provide templates to instill a sense of experimentation in SMEs that connects their business model to a broader array of urban processes, many faced by sustainability challenges and climate pressures. Business models for a good Anthropocene will need to equip businesses to maintain new experimentation and decision-making capacities based on uncertainty, opportunity, and collaborative approaches. Sustainability science offers abundant examples of the shape that such experimentation can take, and it is crucial that these experiments are comprised of both an intervention as well as some mechanism to gather empirical data that demonstrates the outcomes or outputs of the experiment (Luederitz et al. 2017; Caniglia et al. 2017).

Transdisciplinary research approaches can enable collaboration with SMEs and support experimentation that introduces radical and innovative ideas. This includes testing new models and practices with external actors with more experience, knowledge or seeking to find solutions to shared problems (Hirsch Hadorn et al. 2006)–including policy blockages, financial constraints, or technological resources–where scholars can work with managers to embed a science approach to testing and failure, recognizing lessons and developing new tools for sustainability practice (Lang et al. 2012).

### Establishing new hierarchies

Agile and innovative responses to sustainability challenges require a dynamic organizational system which provides opportunities for individuals, regardless of their position in the hierarchy of the organization, to lead on different sustainability initiatives. This implies establishment of decision-making processes that identify and promote inclusive forms of leadership. The accepted decision-making processes mimic and operationalize profit-seeking values that are embedded in a traditional model of economic performance. Establishing a new culture that embodies transformative social and environmental values requires space for individuals to foster new practices. For example, establishing sustainable development partnerships and corporate social responsibility initiatives can become a steppingstone (Moore et al. 2015). Ultimately, however, the lens must shift from the firm-centered pursuit of a social license or cutting financial costs to the integration of strategic mechanisms for building capacities within the enterprise, and among the community partners.

Hardened hierarchies in an organization can become barriers that silo or stall efforts to shift the organization’s culture towards a sustainable pathway. The origin of new practices or emerging values might be introduced by an employee, temporary partner, or collaborator. The organization must be willing and capable of identifying ideas that might require placing those ideas (or the individuals who generated them) at the top of the hierarchy (Kurucz et al. 2014) supporting them with increased budget allocations or time in meetings, and even pursuing advocacy and education on specific sustainability solutions among their clients and stakeholders. For example, a construction waste removal company in Vancouver focuses on construction waste diversion and circular economy practices. Their core business income flows from the sale of reusable materials from local construction sites, but their business priority includes educating and training construction companies, lobbying and advocating for city regulation to adapt municipal landfills and recycling stations to manage diverse waste streams from construction and mobilizing local partners to grow a circular system. The business hierarchies and model has expanded beyond a narrow bottom line approach.

The individual enterprise can reveal and examine inconsistencies wherein actions or values in one area might not be consistent with activities in another area of the organization, or the deeper purpose of the firm. A change in hierarchies can support the examination of all processes, relationships, and behaviours through a new lens, to work towards a shared sustainability objective. Business models that are responsive to the Anthropocene require bold approaches to structuring their operations and a strategic approach underlined by a systemic understanding of sustainability challenges. Genuinely transformative business models will begin to recognize and accept losses associated with change, to seek out avenues that shape individual aspirations and reorient the purpose of enterprises to fulfill fundamental needs in their communities.

The efforts to learn and contribute to the local context, institutionalizing co-production and experimentation would open the need for SMEs to codify and apply new forms of knowledge, integration of business strategies informed by both the success and failures of engaging in sustainability and climate actions. This requires that the decision-making process shift accordingly to allow the organization to attain and deliver the value generated from these new building blocks, much like the traditional business model components provide value for the organization. The re-organization of decision-making processes, investment priorities, and strategic pursuits require hierarchies to change within organizations. Approaches focused exclusively on the traditional financial bottom line, which might underlie many sustainability initiatives, is not a tenable approach even for those enterprises that might continue to seek specific economic or reputation value narrowly. Transdisciplinary research can support the messy and challenging work of uncovering and shifting paradigms and hierarchies in business environments. For instance, researchers can help businesses develop and articulate long-term visions of socio-environmental change, and examine the ways that existing decision-making hierarchies or power imbalances collide with these visions. The goal of this process is not only to shift power and decision-making structures within the firm, but also to prioritise investments, new processes, and resources to address future scenarios (Kläy et al. 2015).

## Nourishing and acting on imagination and play

Unsustainable development patterns and socio-economic marginalization are reproduced and reinforced by institutions, discourses, and the widely dominant structure and function of the private sector. Unearthing the leverage points that allow for non-linear shifts in these pathways is a central task in pursuing sustainability (Abson et al. 2017; Meadows 2008). To accelerate transformative shifts toward sustainability, the formation of new capabilities built on practices that nourish imagination, creativity, and play are foundational for collective action on sustainability and resilience (Pereira et al. 2019; Moore and Milkoreit 2020). While fixed routines and processes in firms reinforce organizational culture, they also allow organizations to establish momentum, language and rhythms belonging to their codes of practice (Feldman 2000). Indeed, organizational culture can stifle the adoption of new practices or prevent the emergence of novel or radical ideas that potentially could enhance the firm’s ability to deliver on different sustainability domains (Ramirez et al. 2014). Therefore, recognizing and actively designing interventions to work with SMEs to introduce or test new methodologies that shift the power dynamics, engage individuals in creative activities free from constraints of time or judgement based on business objectives, will contribute to creating a nourishing environment for employees to act on imagination of new solutions (Kajzer and Walinga 2017). Transdisciplinary researchers can actively establish new partnerships that nourish imagination in organizations to create nimble and adaptive enterprises capable of drawing on their existing pool of expertise and knowledge in new ways.

We find examples of practices in Fab Labs, maker spaces, urban labs, and creative hubs to suggest how imagination and play can become foundational practices for transformation in broader private sector activity systems. For example, to “curate happenstance” as a critical process for supporting talent development and instilling a sense of multi-disciplinary openness or providing access to resources for scientists, artists, educators, and amateurs to play, mentor and collaborate. These initiatives offer lessons on how imaginative and play-driven activities can help create enabling ecosystems across industries and scales for organizations to address sustainability problems and enable a collaborative environment for actors invested in local transformation to engage in the co-production of local sustainability solutions. The types of practices that we find in these creative spaces illustrate how entrepreneurship and creativity, cooperation and imagination led processes can lead to concrete sustainable solutions. These range from providing local micro-manufacturing capacities to more strategic digital and creative art enterprises where cooperation and creativity are the driving force behind collaborations, including open spaces and inviting environments for individuals to explore new ideas. These spaces can provide examples of different approaches that sustainability scientist, business managers and practitioners can consider engaging employees, suppliers, and partners to work in solving problems or proposing new solutions. The design of transformative spaces (Pereira et al. 2018a; b) requires, similarly creating environments for creative problem solving across all hierarchies of the company and providing opportunities for employees or partners to work collaboratively, calls for different norms, values, and physical spaces, such as the ones described in the creative hubs and laboratories.

The potential for happenstance, creativity, and unstructured exploration of new ideas can reveal important opportunities to enhance social and ecological resilience. These features of transformative models are oriented towards cultivating multi-disciplinarity, the establishment of a shared language amongst individuals with different forms of knowledge, experiences, and capacities, and providing a nurturing environment for radically imaginative future visions (Burch et al. 2019).

These are practices are central to resilience building, both at the organizational and community scale, where a rhythm (Lefebvre 1992) of collaboration for shared sustainability goals will be critical to sustaining collaborative work amongst partners. A creative space is one in which organizational norms allow for such exploration, with minimum structure and expectations, but curating a pathway for sustainability thinking to thrive (Carlsson et al. 2015). The development and expansion of skill sets based on exercising individual creativity and imagination can help organizations navigate different complexity characteristics of rapid environmental or social change.

The configuration of transformative business models must allow space for solutions based on imagination. Transdisciplinary research can cross pollinate different industries to transfer practices and methodologies (Taylor et al. 2020) that assist in the creation of transformative business models. Examples draw upon the unique capacities within the digital arts and creative industries and include introducing games that facilitate mapping of risk and resilience (Taylor et al. 2020) or developing new visions of sustainability (Vervoort et al. 2010; Vervoort 2019). Finally, nourishing ‘play’ as a practice in everyday work to allow for unstructured time, experimentations, testing, and instilling a sense of freedom in the organization for individuals' ideas or actions is fundamental for adults to think through sustainability problems.

## Conclusions

The limits of unsustainable growth, rising inequality, risk and losses in the biosphere point to the urgent need for transformation. The bottom-line logic embedded in private enterprise, driving most national economies and shaping activities at the local scale, requires rapid support to accelerate the proliferation of business models suitable for the challenges of the Anthropocene.

Indeed, it is necessary for transformative models to emerge and replicate at a pace that ensures significant progress on local development objectives. This requires a set of capabilities which have not been widely exhibited by organizations in the private sector, or have been limited to a few industries in the technology or creative sectors. An enabling ecosystem (cf. Biggeri et al. 2017) requires policy innovations, financial support, and tax incentives that foster experimentation, promote close collaborations, and deepen coordination among different actors.

We recognize the emerging diversity of private sector business models and the multiplicity of entrepreneurial efforts that illustrate alternative values in business functions. We argue that spreading and deepening these new business models might not conform to existing organizational hierarchies but will be indispensable features of business models in the future. Building the capabilities necessary for private sector actors to change their approach to engaging in profit seeking activities, requires a shift in mindsets and value systems towards consideration of constituencies without a voice (such as future generations and non-human nature). Each building block that we identify here aims to embed a new conduit by which individuals and teams in these organizations can approach decision-making processes, partnerships, and the incorporation of new information into the organizational system. Taken together, these building blocks provide the foundational support that organizations require to eventually leap and accelerate sustainable approaches to economic activities. As more organizations begin to make such leaps, local and regional economies might begin to operate in greater alignment with the needs and challenges faced by their communities.

While transformations remain the exception rather than the rule, and sustainability goals become ever more pressing, it is the task of sustainability scientists to critically assess the real impact and outcome of the sustainability efforts that firms report. Indeed, inclusive, creative, and accelerated change will not occur at the speed needed as long as organizations lack the capabilities, purpose, metrics or desire to shift towards new pathways. We do, however, find emerging evidence of change at different scales, both in small enterprises and larger organizations, or so-called keystone actors in sustainability (Hileman et al. 2020). As we begin to map and identify sustainability patterns, a lattice of positive changes can be observed, not sufficient to conclusively demonstrate transformation, but enough to illustrate how new practices and models can begin to replace some elements of existing regimes’ unsustainable practices.

Helping managers learn and contribute to local communities will pinpoint where new partnerships on specific sustainability problems might be valuable or identify new sources of risk. Likewise, nourishing individuals’ ability to conceive of new practices, and connect them to existing organizational cultures and language in their company is paramount to developing an organization apt for the challenges of the following decades. Therefore, transdisciplinary research approaches must continue its work on nourishing imagination through experimentation and co-production rooted in meaningful trust-building.

As communities envision economies rooted in a much broader, more inclusive, and more sustainable set of values, a renewed sense of purpose for individuals who are part of private sector organizations will help accelerate this shift. A radical departure from existing models is necessary, however, to reconstruct the private sector in a way that encourages curiosity, cooperation, and creativity in individuals, while promoting inclusivity, and creating a culture that actively seeks to address socio-ecological problems.
